# Age and individual differences in visual working memory deficit induced by overload

**DOI:** 10.3389/fpsyg.2014.00384

**Published:** 2014-05-07

**Authors:** Daisuke Matsuyoshi, Mariko Osaka, Naoyuki Osaka

**Affiliations:** ^1^Department of Psychology, Graduate School of Letters, Kyoto UniversitySakyo, Japan; ^2^Department of Integrative Physiology, National Institute for Physiological SciencesOkazaki, Japan; ^3^Department of Psychology, Graduate School of Human Sciences, Osaka UniversitySuita, Japan; ^4^Research Center for Advanced Science and Technology, The University of TokyoKomaba, Japan

**Keywords:** aging, capacity limit, individual differences, object recognition, visual working memory

## Abstract

Many studies on working memory have assumed that one can determine an individual's fixed memory capacity. In the current study, we took an individual differences approach to investigate whether visual working memory (VWM) capacity was stable irrespective of the number of to-be-remembered objects and participant age. Younger and older adults performed a change detection task using several objects defined by color. Results showed wide variability in VWM capacity across memory set sizes, age, and individuals. A marked decrease in the number of objects held in VWM was observed in both younger and older adults with low memory capacity, but not among high-capacity individuals, when set size went well beyond the limits of VWM capacity. In addition, a decrease in the number of objects held in VWM was alleviated among low-capacity younger adults by increasing VWM encoding time; however, increasing encoding time did not benefit low-capacity older adults. These findings suggest that low-capacity individuals are likely to show decreases in VWM capacity induced by overload, and aging exacerbates this deficit such that it cannot be recovered by simply increasing encoding time. Overall, our findings challenge the prevailing assumption that VWM capacity is fixed and stable, encouraging a revision to the strict view that VWM capacity is constrained by a fixed number of distinct “slots” in which high-resolution object representations are stored.

## Introduction

It has long been assumed that humans can concurrently hold 3 to 4 objects in visual working memory (VWM) (Luck and Vogel, 1997; Cowan, 2001). However, this number is not absolute and is subject to change based on individual differences and the memoranda used (Alvarez and Cavanagh, 2004; Vogel and Machizawa, 2004). By assessing individual differences in memory capacity, VWM studies have suggested that attentional control efficiency is closely related to the particular memory capacity of each individual (Vogel et al., 2005; Vogel and Awh, 2008).

The assumption that VWM capacity is fixed and stable within individuals given identical memoranda is critical to VWM studies using an individual differences approach; however, direct evidence for this stability across set sizes and age has not been provided. Previous studies have sporadically reported a decline in VWM capacity at large set sizes (Chee and Chuah, 2007; Xu, 2007; Cusack et al., 2009; Edin et al., 2009). Using a change detection paradigm in which participants are required to detect a change between sample and test displays, these studies found a decrease in VWM capacity with overload (i.e., set sizes were large enough to exceed the canonical number of the VWM capacity limit). However, because this decline is outside the focus of most studies, the mechanisms for the decline remain largely unknown. Cusack et al. (2009) directly investigated this decline in VWM capacity. Participants performed two tasks: one was a change detection task using letters, and the other was a whole report task in which participants were required to report all the letters they could remember. The authors found that a decrease in VWM capacity at large set sizes occurred only during change detection task, and the decline was more severe among participants with lower fluid intelligence, as measured by Cattell's (1973) culture fair test. Based on these findings, it was suggested that a change detection paradigm, but not a whole report paradigm, imposes maladaptive encoding strategies on participants (particularly those with lower intelligence) at large set sizes, thereby decreasing the number of objects held in VWM. The change detection paradigm might encourage participants to hold a visual snapshot of an entire display while the whole report paradigm might encourage covert naming of letters and lead to more selective encoding. However, this aforementioned study had an older sample, including participants over the age of 60 (mean age = 47 years), aging could be the predominant driver of these effects. Considering the diverse influences of aging on working memory (Cowan et al., 2006; Logie and Maylor, 2009; Brockmole and Logie, 2013), further investigation is warranted.

In addition, individual differences in VWM capacity might affect declines in performance due to overload. Individual differences in memory capacity are known to correlate with efficient control of representations in VWM (Vogel et al., 2005; Luck and Vogel, 2013). It has been shown that low-capacity individuals are more likely to exhibit inefficient or maladaptive object encoding than high-capacity individuals. However, whether individual differences in memory capacity likewise affect the degree of the decline in VWM capacity has not been established.

In the present study, we conducted two experiments in which younger and older adults were divided into separate groups in order to examine the influence of age and individual differences on the decline in VWM capacity at large set sizes. We hypothesized that a markedly larger decline would be observed among older adults as compared to younger adults, and this decline would also be related to individual differences in memory capacity. Such results would suggest that VWM capacity is unstable and vulnerable to aging and the memory capacity of each person. Furthermore, these results would add to the debate on the constraints in VWM capacity (i.e., the fixed-resolution slot vs. flexible-resource models, see Luck and Vogel, 2013; Ma et al., 2014). The flexible-resource model posits that resource allocation becomes critical with large set sizes, because there are few resources to be allocated to each item (see Bays and Husain, 2008; van den Berg et al., 2012). Meanwhile, the slot model posits that individuals store a high-resolution representation of a subset of objects irrespective of set size (see Zhang and Luck, 2008; Barton et al., 2009). The existence of a decline in performance with overload would support the flexible-resource, rather than the slot model of VWM capacity.

## Experiment 1

### Methods

#### Participants

All participants had normal or corrected-to-normal visual acuity and normal color vision, and reported no history of psychiatric or neurological disorders. All older participants were administered the Hasegawa Dementia Scale-Revised test, and were found to be cognitively sound (mean score: 28.8, range: 24–30). Each participant gave written informed consent after being apprised of the procedure.

Forty-five younger adults and 45 older adults participated in Experiment 1. Data for one younger adult with a visual disability and three older adults who misunderstood the task rules and/or failed to perform the task correctly were excluded from the analysis. Data from the remaining 44 younger adults (15 females; mean age: 21.3 years, range: 18–27 years) and 42 older adults (19 females; mean age: 70.3 years, range: 62–78 years) were analyzed.

#### Materials and procedure

**Figure 1:**
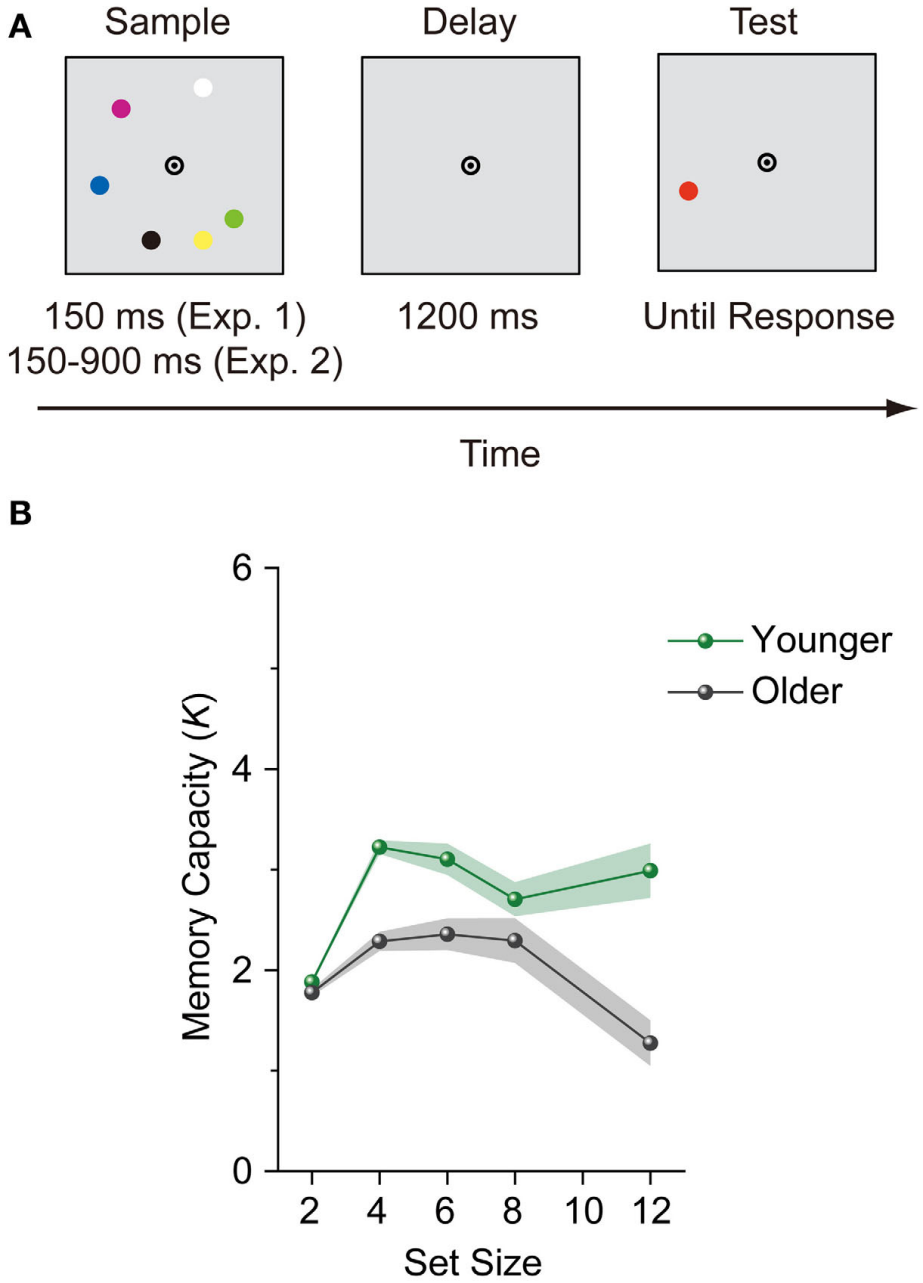
**(A)** An example trial for the change detection task. Sample display duration was different between Experiments 1 and 2. **(B)** Mean memory capacity for younger and older adults in Experiment 1. Shaded regions denote ±1 s.e.m.

All stimuli were presented on a gray background. Each trial was initiated by pressing a button. After presenting a central fixation (bull's eye) display of a randomized duration (500–1000 ms), a sample display containing two, four, six, eight, or twelve 0.7° discs selected randomly from a set of seven colors (red, green, blue, yellow, violet, black, and white) was presented for 150 ms. Color repetition was allowed, with the constraint that the same color could not be repeated more than twice on each display. The colored discs were located 2.3° from the fixation point and placed on the 12 possible apexes of an imaginary dodecagon. A 1200-ms blank interval was presented following the sample display, and a colored probe disc (test display) was then presented in one of the previously occupied locations (Figure 1A). Each set size was presented 48 times (240 trials in total). The color of the probe was matched to the sample display for half of the trials, but not for the other half. Participants were required to indicate whether a change had or had not occurred between the sample and test displays while fixating on the central bull's-eye. Accuracy was emphasized over speed. Memory capacity was estimated from Cowan's *K* formula: *K* = (H – F) × S, where *K* is the VWM capacity, H is the hit rate, F is the false alarm rate, and S is the display set size (Cowan, 2001).

### Results and discussion

To evaluate the stability of VWM capacity across set sizes, we performed a repeated-measures ANOVA on participants' *K* scores with age group (younger and older adults) as a between-subjects factor and set size (4, 6, 8, and 12) as a within-subjects factor. Set size 2 was not included in this ANOVA, because this set size does not reach the canonical capacity limit of VWM (about 3 to 4 objects) (Luck and Vogel, 1997; Cowan, 2001) and therefore is not suitable for assesing stability in VWM capacity. The main effects of age group [*F*_(1, 84)_ = 28.178, *p* < 0.0001, η^2^_*p*_ = 0.251] and set size [*F*_(3, 252)_ = 7.345, *p* = 0.0001, η^2^_*p*_ = 0.080], and the interaction between age group and set size [*F*_(3, 252)_ = 6.732, *p* = 0.0002, η^2^_*p*_ = 0.074] were significant. *K*s did not vary significantly from set sizes 4 to 12 among younger adults [*F*_(3, 129)_ = 2.073, *p* = 0.107, η^2^_*p*_ = 0.046] (Figure 1B), while *K*s decreased significantly from set sizes 4 to 12 among older adults [*F*_(3, 123)_ = 12.571, *p* < 0.0001, η^2^_*p*_ = 0.235]. A large decrease in *K*s was observed specifically at set size 12. Although older adults could remember a maximum of 2.35 items at set size 6, this was reduced to 1.27 items at set size 12.

**Figure 2:**
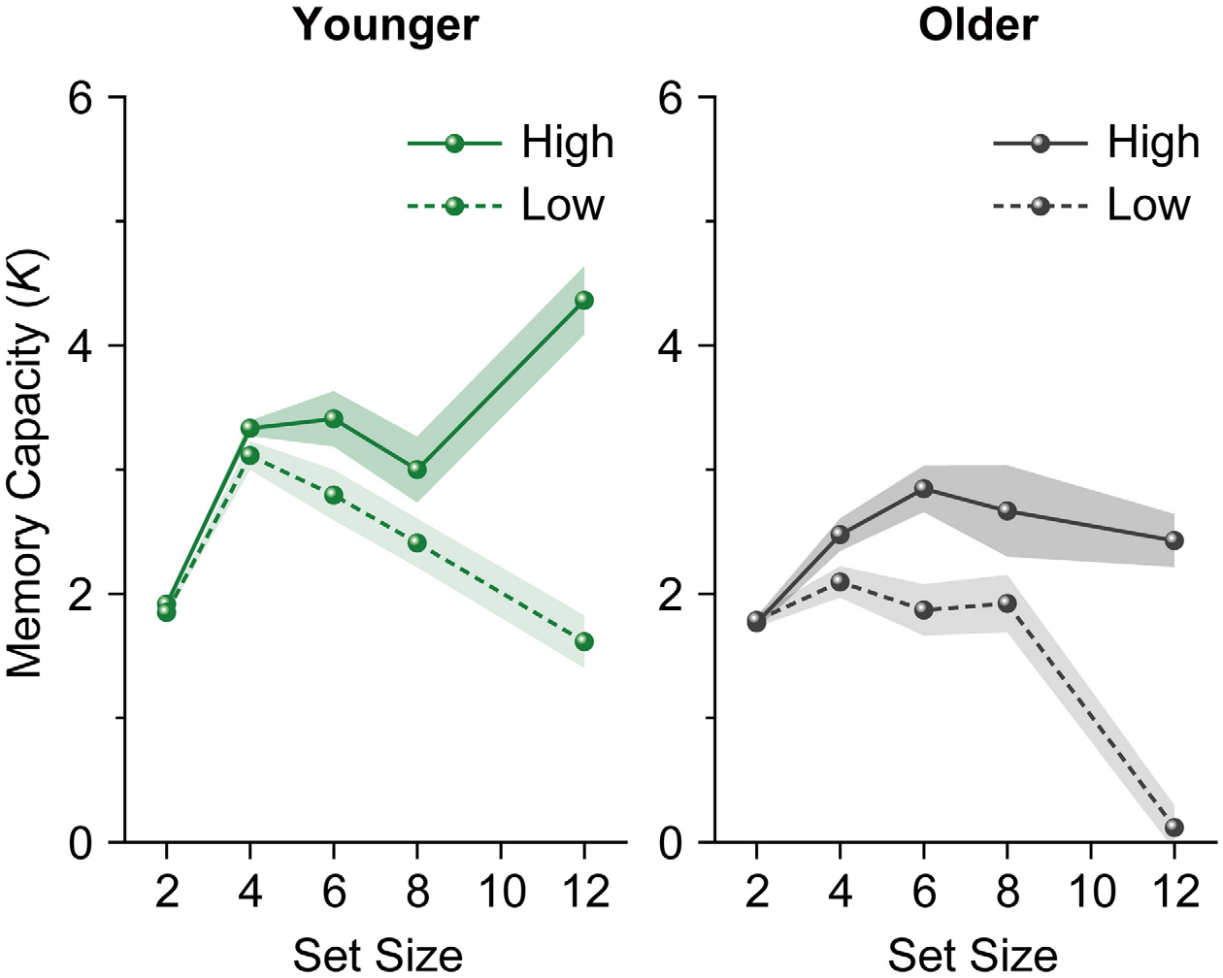
**Mean memory capacity for younger and older adults divided across high and low memory capacity groups in Experiment 1**. Shaded regions denote ±1 s.e.m.

The plateau found in the memory capacity slope does not necessarily reflect stability in VWM capacity within individuals. Given the increase in individual differences with an increase in set size, as is clearly shown by the error bars, this plateau might reflect the net result of high and low memory capacity individuals (see also Edin et al., 2009). Thus, we divided participants into high and low memory capacity groups using a median split of their memory capacity (*K*) at set size 12 (Figure 2), and performed a repeated-measures ANOVA on participants' *K*s (4, 6, 8, and 12) as a within-subjects factor separately for each age and capacity group. We found a decrease in *K*s among both younger and older adults with low capacity [younger adults, *F*_(3, 63)_ = 13.936, *p* < 0.0001, η^2^_*p*_ = 0.399; older adults, *F*_(3, 60)_ = 28.161, *p* < 0.0001, η^2^_*p*_ = 0.585]. *K*s increased among high-capacity younger adults [*F*_(3, 63)_ = 9.621, *p* < 0.0001, η^2^_*p*_ = 0.314], while an asymptote of *K*s was observed among high-capacity older adults (*F* < 1).

These results showed that when set size exceeded a participant's capacity limit, a significant decrease in the number of items remembered was evident among older adults (especially those with low capacity). In addition, although younger adults appeared to hold a certain number of items in VWM, this resulted from averaging high and low capacity individuals. A similar decrease in VWM capacity was observed among low-capacity younger adults at large set sizes, while high-capacity younger adults showed an increase in VWM capacity as set size increased.

## Experiment 2

In Experiment 1, we found age and individual differences in the susceptibility of VWM capacity to overload, using a change detection task with canonical time parameters (e.g., Luck and Vogel, 1997; Todd and Marois, 2004; Matsuyoshi et al., 2012). However, the short duration (150 ms) of sample displays, which was fixed across set sizes, might have been too demanding on VWM encoding, particularly at large set sizes (e.g., 12.5 ms per object at set size 12). This temporal constraint when encoding objects might have caused the decrease in VWM capacity. In addition, although Cusack et al. (2009) suggested that the decline due to overload might occur during the encoding phase, direct behavioral evidence is lacking. To address these issues, we elongated the duration of sample displays (75 ms per object) in Experiment 2, so that the encoding time for each object sufficiently exceeded the known rate of VWM consolidation (50 ms per object) (Vogel et al., 2006).

### Methods

#### Participants

Seventeen younger adults and 14 older adults participated in Experiment 2. Data for one younger adult who failed to perform the task correctly were excluded from the analysis. Data from the remaining 16 younger adults (eleven females; mean age: 21.0 years, range: 19–25 years) and 14 older adults (six females; mean age: 71.2 years, range: 66–78 years) were analyzed.

#### Materials and procedure

All materials and procedures were identical to Experiment 1, except that the duration of the sample display was scaled relative to the memory set size (150, 300, 450, 600, and 900 ms for set size 2, 4, 6, 8, and 12, respectively).

### Results and discussion

**Figure 3:**
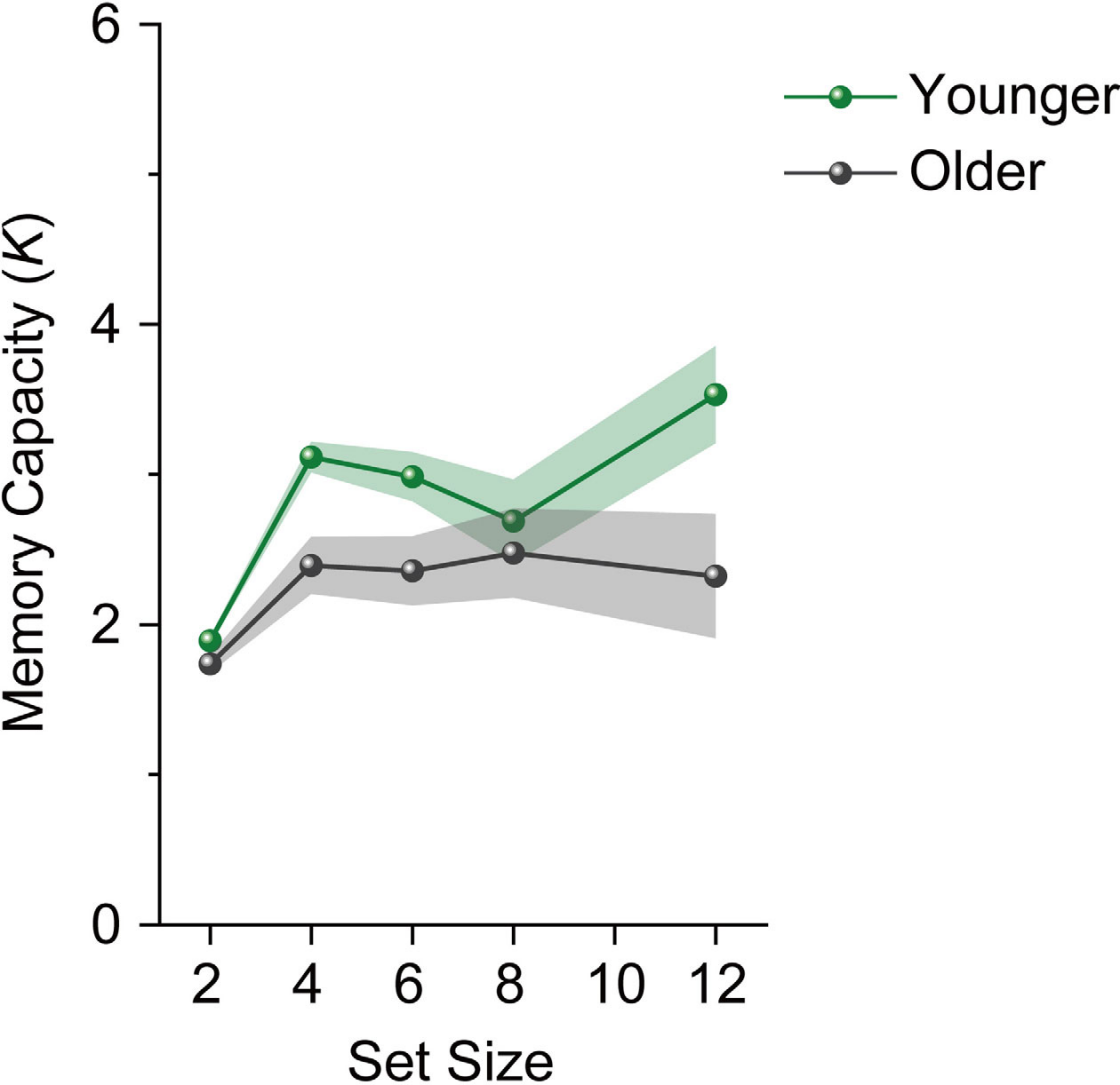
**Mean memory capacity for younger and older adults in Experiment 2**. Shaded regions denote ±1 s.e.m.

**Figure 4:**
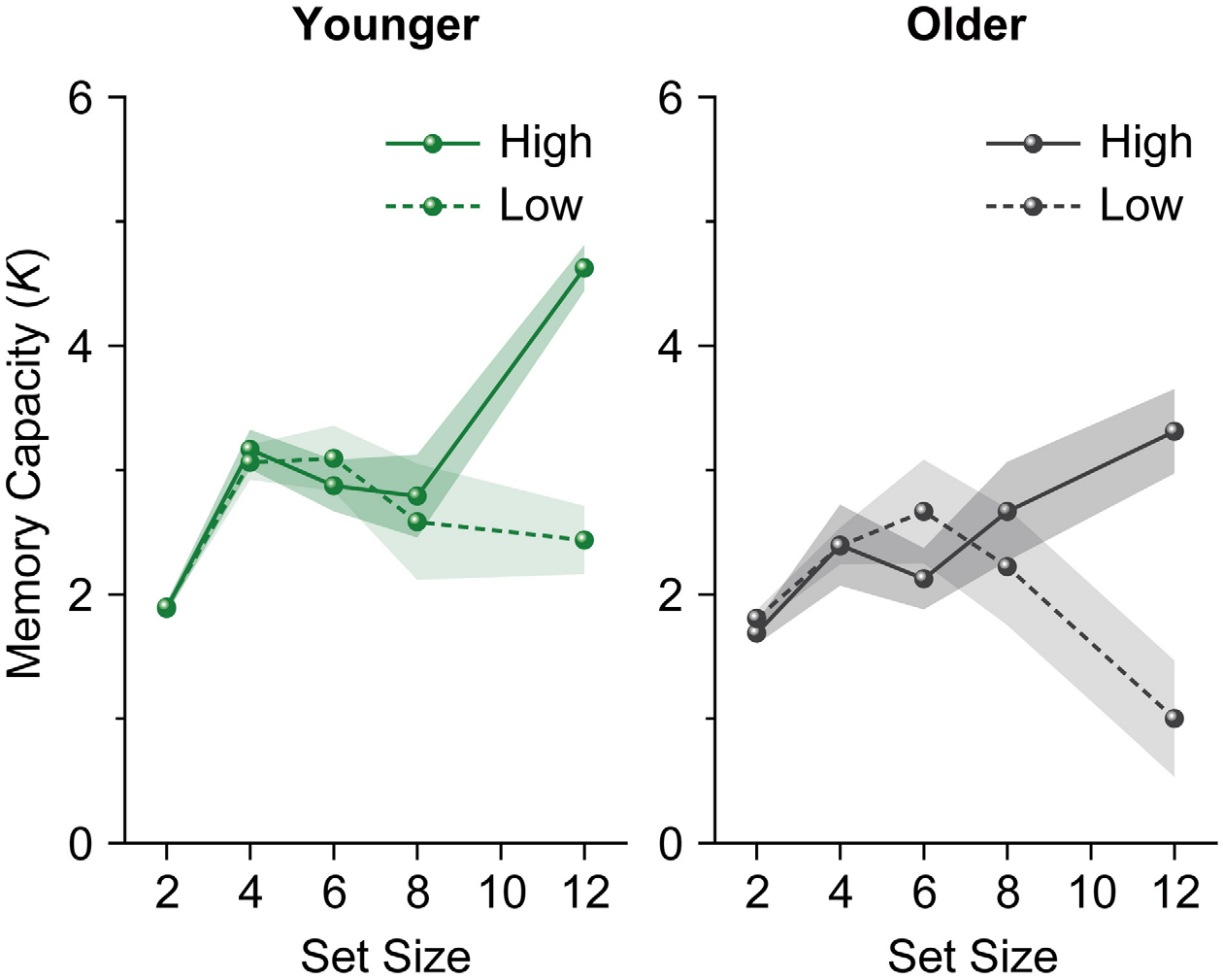
**Mean memory capacity for younger and older adults divided across high and low memory capacity groups in Experiment 2**. Shaded regions denote ±1 s.e.m.

We performed a repeated-measures ANOVA on participants' *K* scores with age group (younger and older adults) as a between-subject factor and set size (4, 6, 8, and 12) as a within-subject factor. The analysis revealed a significant main effect of age group [*F*_(1, 28)_ = 10.658, *p* = 0.003, η^2^_*p*_ = 0.276]. However, the main effect of set size (*F* < 1), and the interaction between age group and set size [*F*_(3, 84)_ = 1.339, *p* = 0.267, η^2^_*p*_ = 0.046] were not significant. *K*s for younger adults reached an asymptote above set size 4 [*F*_(3, 45)_ = 2.228, *p* = 0.098, η^2^_*p*_ = 0.129] (Figure 3). Unlike in Experiment 1, *K*s for older adults reached an asymptote in Experiment 2 (*F* < 1). We then performed a repeated-measures ANOVA on participants' *K*s with set size (4, 6, 8, and 12) as a within-subject factor separately for each age and capacity group. Both high-capacity younger and older adults showed an increase in *K*s across set sizes [younger adults, *F*_(3, 21)_ = 12.643, *p* < 0.0001, η^2^_*p*_ = 0.644; older adults, *F*_(3, 21)_ = 3.169, *p* = 0.046, η^2^_*p*_ = 0.312] (Figure 4). Low-capacity older adults showed a decrease in *K*s [*F*_(3, 15)_ = 5.079, *p* = 0.013, η^2^_*p*_ = 0.504], while low-capacity younger adults reached an asymptote [*F*_(3, 21)_ = 1.199, *p* = 0.335, η^2^_*p*_ = 0.146].

**Figure 5:**
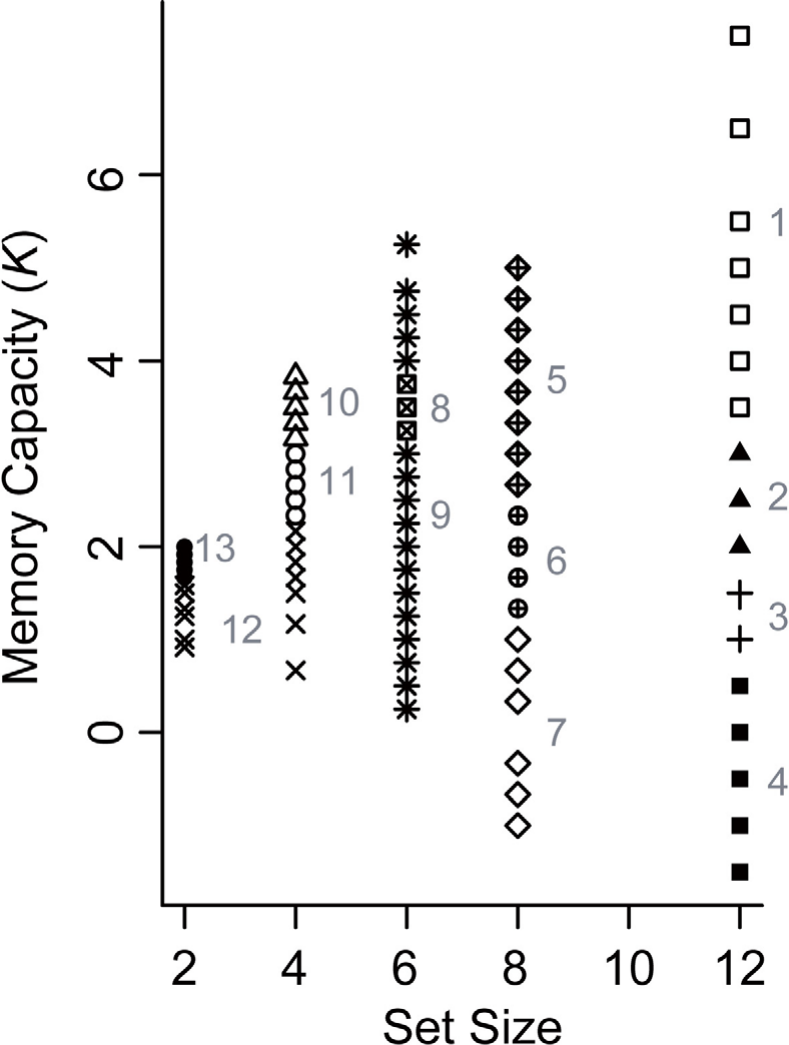
**Latent memory capacity clusters obtained from Gaussian finite mixture modeling using pooled data from Experiments 1 and 2**. Different shapes indicate different clusters. The numbers in gray indicate the cluster ID.

**Figure 6:**
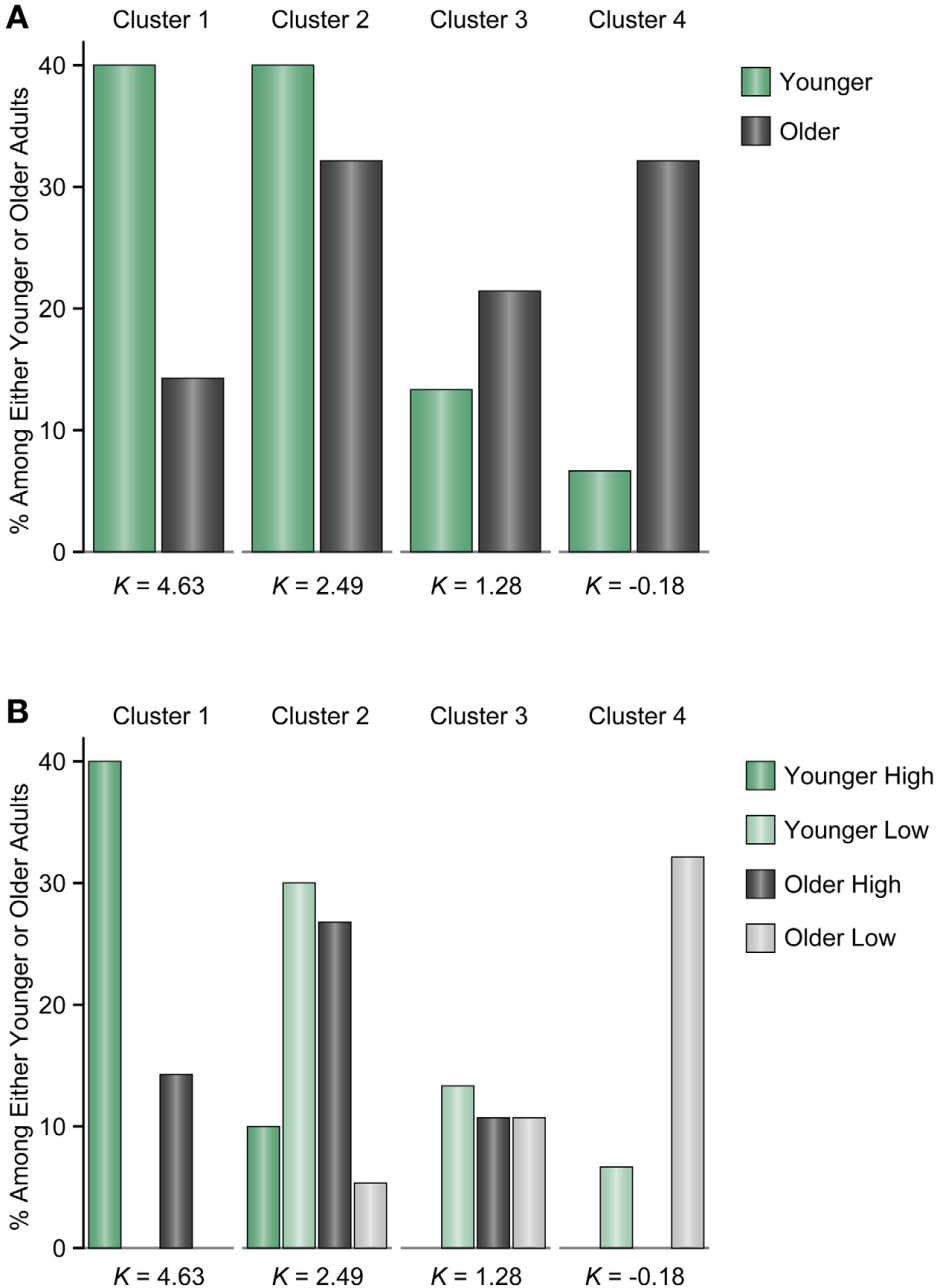
**(A)** The proportion of younger and older adults among the four clusters at set size 12. **(B)** The proportion of younger and older adults divided across high and low memory capacity groups among the four clusters at set size 12. The high and low memory capacity groups were derived from each experiment. The *K*-values under the bars indicate mean memory capacity for each cluster.

To evaluate the differences between younger and older adults in more detail, we performed a Gaussian finite mixture modeling (McLachlan and Peel, 2000), which is a modeling procedure for representing latent clusters in data, using pooled data from Experiments 1 and 2 [*n* = 580 (116 participants × 5 set sizes)]. This mixture modeling, using an expectation—maximization algorithm, enabled us to compare the differences between younger and older adults in the latent memory capacity cluster to which they belong. This analysis converged on a model (log likelihood = −1429.807, Bayesian Information Criterion value = −3184.129) with 13 clusters (1, 1, 2, 2, 3, and 4 clusters at set size 2, between set sizes 2 and 4, and at set sizes 4, 6, 8, and 12, respectively) (Figure 5). It is, however, notable that this large number of clusters was probably induced by the discrete nature of the x-axis (i.e., set sizes 2, 4, 6, 8, and 12) compared to the relatively continuous nature of the y-axis (i.e., Cowan's *K*) in the present study. It is likely that the discontinuous characteristics of set sizes made it hard to find “across-set-size” clusters. Thus, we focused on the four clusters at set size 12, which are most indicative of a decline in VWM capacity, and found that the proportion of younger and older participants remarkably differed between the higher (clusters 1 and 2) and lower *K* clusters (clusters 3 and 4) [*χ*^2^_(1)_ = 14.134, *p* = 0.0002, φ = 0.349] (Figure 6). Fifty four percent of older adults belonged to the lower memory capacity clusters, while only 20% of younger adults belonged to these clusters, which indicated a stronger susceptibility of VWM capacity to overload among older adults. Note also that the negative value of cluster 4 does not mean that memory capacity is actually negative, but that it is essentially zero. This negative value was induced by the small number of participants (two younger, and five older adults in Experiment 1, and one older adult in Experiment 2) who showed negative *K*-values at set size 12. Decreases in VWM capacity remained significant even if negative *K*-values were recoded as zero [low-capacity younger adults in Experiment 1, *F*_(3, 63)_ = 13.623, *p* < 0.0001, η^2^_*p*_ = 0.393; all older adults in Experiment 1, *F*_(3, 123)_ = 10.638, *p* < 0.0001, η^2^_*p*_ = 0.206; low-capacity older adults in Experiment 1, *F*_(3, 60)_ = 23.658, *p* < 0.0001, η^2^_*p*_ = 0.542; low-capacity older adults in Experiment 2, *F*_(3, 15)_ = 4.960, *p* = 0.014, η^2^_*p*_ = 0.498].

A decline in VWM capacity at large set sizes was observed among both younger and older adults with low capacity in Experiment 1. By increasing the encoding time for objects in Experiment 2, we found that low-capacity younger adults recovered from the deficit whereas low-capacity older adults did not. Our results indicate that encoding time plays a critical role in the decline; however, increased encoding time might not be sufficiently effective for low-capacity older adults, as they did not show any recovery from the deficit even when additional time was provided for VWM encoding.

## General discussion

The current study investigated the stability of VWM capacity, as measured by a change detection task, across age and set sizes. A decline in VWM capacity at large set sizes was observed in both younger and older adults with low memory capacity; however, only younger adults recovered from this decline when additional time was provided for object encoding. These results suggest a marked age-related deficit in VWM with overload and that stability in VWM capacity, as estimated by a change detection paradigm, depends on the age and memory capacity of each individual.

Cusack et al. (2009) suggested that VWM capacity estimated by a change detection paradigm would likely induce a decrease at large set sizes, whereas VWM capacity estimated by a whole report paradigm would not. However, the relatively higher age of their participants rendered their assertions inconclusive. A decline with overload might reflect the effects of aging (see Cowan et al., 2006; Logie and Maylor, 2009; Brockmole and Logie, 2013) above and beyond the differences in paradigms. By examining performance during a change detection task separately in younger and older adults, the present results showed that only older adults exhibit a decline at large set sizes (based on group averages). Although it is difficult to dissociate the effects of aging-related declines in fluid intelligence (McArdle et al., 2002), our results suggest that this decline is closely associated with general aging.

The stability of VWM capacity in younger adults manifested only when data were *averaged* across individuals. Our analysis assesing individual differences revealed that a decrease was also observed among younger adults with *low capacity*. These results suggest that a decline at large set sizes depends not only on age, but also on each person's memory capacity. Experiment 2 showed that older adults with low capacity did not recover from a decline in VWM capacity even when additional time was provided for encoding, which was different from what was observed among younger adults with low capacity. Low-capacity older adults were more likely to show a marked deficit in VWM as compared to other groups (see also Jost et al., 2011).

Although the exact mechanisms underlying the deficits among low-capacity individuals are not fully known, we propose three plausible factors. First, these individuals might fail to control their attention when selecting objects within their VWM capacity limit while overloaded (top-down control view). They might attempt to hold all objects (e.g., Cusack et al., 2009) even though the set size exceeds their individual capacity limit, resulting in a capacity “overflow” in VWM. Second, these individuals might be slow to recover from attentional capture (attentional capture view). Attention is captured and distributed across objects after sample display onset; however, low-capacity older adults might be especially slow to recover from this attentional capture (e.g., Fukuda and Vogel, 2011) and fail to concentrate VWM capacity on a limited number of objects. Finally, these individuals might just need more time to consolidate objects in VWM (slow encoding view). The rate of object encoding among older adults might be slower than among younger adults, and the presentation rate of objects in the present study (75 ms per object) might have been sufficient for younger (e.g., Vogel et al., 2006), but not for older adults. These proposals are not mutually exclusive, and the cause for a decline might be different across individuals; in any case, deficits in VWM among older adults might be more severe than among younger adults, such that the deficit cannot be recovered by simply increasing the encoding time.

It is also important to note that performance among high-capacity individuals improved with an increase in set size (high-capacity younger adults in Experiments 1 and 2, and high-capacity older adults in Experiment 2). This improvement might simply reflect these individuals' higher memory capacity or might be partly caused by the nature of our analysis, which divided participants into high and low capacity groups using performance at set size 12 and then performed a repeated-measures ANOVA with set sizes 4–12. The ANOVA used was non-independent and circulatory, and is biased to obtain a significant effect of set size when high-capacity individuals are the subjects of analysis (see Baker et al., 2007; Simmons et al., 2007; Kriegeskorte et al., 2009; Vul et al., 2009). In other words, there is a chance this analysis inflates our assessment of memory capacity because of an increase in maximum allowable *K* scores with an increase in set size (i.e., one cannot obtain a *K* score that is higher than the set size). However, we did not find performance improvement among high-capacity older adults when encoding duration was limited (in Experiment 1). Moreover, although there was a bias to observe a significant improvement in performance at large set sizes among high-capacity individuals, there is no explicit reason that low-capacity individuals exhibit a decline in memory capacity at large set sizes. If VWM capacity is fixed and stable across set sizes and individuals, the *K* scores of low-capacity individuals should exhibit a plateau at lower *K* scores; however, that was not always the case. Thus, although our results with high-capacity individuals could be affected by the nature of our analysis, results with low-capacity individuals are free from this bias and are considered valid.

## Conclusion

Previous working memory studies using an individual-differences approach have allocated each individual to a certain score regarding his/her memory capacity, investigating the relationships between those working memory scores and broad cognitive abilities (Vogel and Awh, 2008; Luck and Vogel, 2013). However, it is largely unknown whether humans can always maintain a certain number of objects within their VWM capacity limit. Results of the present study demonstrate the instability of VWM capacity, as assessed with a change detection task, based on factors related to age and overload. We found a decrease in the number of items held in VWM when low-capacity and/or older individuals are required to remember several objects that exceeded their capacity limit. Our findings challenge the prevailing assumption that VWM capacity is stable across set sizes and that we can allocate a particular memory capacity score to each individual.

Furthermore, these findings add to the ongoing debate whether VWM capacity is constrained by a fixed number of “discrete slot” or by a limited “flexible resource” that is distributed among items in memory (Luck and Vogel, 2013; Ma et al., 2014). Although the slot-based model predicts that humans should always store a high-precision representation of a fixed number of simple objects (see Zhang and Luck, 2008; Barton et al., 2009), we found that low-capacity and/or older individuals showed a decrease in VWM capacity with overload. These findings are consistent with the flexible-resource model, which predicts that fewer resources are allocated to each object under overload (Bays and Husain, 2008; van den Berg et al., 2012). However, because the exact mechanisms for the deficit remain unclear and may be multifactorial, our findings do not completely reject the slot model. Nevertheless, we suggest that our results at least necessitate a revision or unequivocal delineation of the slot model under high load conditions, and support the flexible-resource model more so than the slot-based model in its current form.

## Author contributions

Daisuke Matsuyoshi designed the study, collected the data, and analyzed the data. All authors contributed to interpretation of the data and drafting this manuscript.

### Conflict of interest statement

The authors declare that the research was conducted in the absence of any commercial or financial relationships that could be construed as a potential conflict of interest.
